# Acquired pure red cell aplasia after severe acute respiratory syndrome corona virus 2 infection: a case report

**DOI:** 10.1186/s13256-022-03545-x

**Published:** 2022-10-19

**Authors:** Imane Karrat, Hicham Eddou

**Affiliations:** 1 Sidi Mohamed Ben Abdellah University, Faculty of Medicine and Pharmacy, Fez, Morocco; 2 Department of Clinical Haematology, Moulay Ismail Military Teaching Hospital, Meknes, Morocco; 3 University Hospital Center Hassan II, Fez, Morocco

**Keywords:** COVID-19, Acquired pure red cell aplasia, SARS-CoV-2

## Abstract

**Background:**

Coronavirus disease 2019, caused by severe acute respiratory coronavirus 2, has been responsible, since December 2019, for a severe pandemic resulting in millions of deaths worldwide, and the number is still increasing. Although coronavirus disease 2019 is mostly a respiratory syndrome, it is considered a multisystemic disease and shows clinical diversity with a wide range of manifestations including hematological features.

**Case presentation:**

We present the case of an Arab male, 77 years old, who developed severe anemia 8 weeks after acute infection with severe acute respiratory coronavirus 2. The investigations revealed acquired pure red cell aplasia. Workup for an associated underlying disorder was negative, ruling out secondary causes. The patient received corticosteroids as the standard treatment of primary acquired pure red cell aplasia, and he had a good response to treatment.

**Conclusion:**

This case illustrates that acquired pure red cell aplasia might occur weeks after severe acute respiratory coronavirus 2 infection, suggesting that it might be considered a delayed complication of coronavirus disease 2019. The most relevant hypothesis of the pathogenesis of acquired pure red cell aplasia, in this case, is an immune mechanism triggered by infection with severe acute respiratory coronavirus 2 resulting in interruption of normal erythroid differentiation. We highlight the importance of follow-up care after the acute phase of coronavirus disease 2019 to spot late complications in order to successfully manage the secondary burden of the pandemic.

## Background

Severe acute respiratory syndrome coronavirus 2 (SARS-CoV-2) belongs to the genus Betacoronavirus of the family of coronaviruses. Coronaviruses have crown-like structures and are enveloped and positive-sense single-stranded RNA viruses, with the largest RNA genomes (27–32 kb) among the RNA viruses. They are usually responsible for zoonotic diseases but can be transmitted to humans, likely via animal reservoirs [1].

In December 2019, SARS-CoV-2 caused a large-scale outbreak resulting in a pandemic that rapidly spread all over the world.

SARS-CoV-2 is highly contagious and believed to be transmitted primarily through respiratory droplets, aerosols, and direct contact with contaminated surfaces. SARS-CoV-2 affects the upper and lower respiratory tract, resulting in severe respiratory distress syndrome that is potentially deadly. The most frequent symptoms of coronavirus disease 2019 (COVID-19) are dry cough, fever, and fatigue. In addition to respiratory distress, it can affect a wide range of systems, including renal, cardiovascular and circulatory systems, which explains the diversity of clinical manifestations of COVID-19 [2]. The most severe forms are likely caused by a hyperinflammatory condition leading to multiorgan failure and death [3].

Currently, the number of cases has increased massively, with many studies reporting the clinical manifestations and complications of COVID-19. Hematological manifestations have been reported in multiple studies, and are known to have a prognostic impact. Here we report a case of acquired pure red cell aplasia (PRCA) potentially linked to infection by SARS-CoV-2.

## Case report

We report the case of an Arab male patient, 77 years old, known to have arterial hypertension for which he was not taking any medications. He had no personal or familial history of anemia.

In November 2020, he was admitted for acute onset of dyspnea and episodes of productive cough, fever, and fatigue, with no chest pain or gastrointestinal symptoms. On examination, the patient appeared in respiratory distress. He was weak and fatigued. His temperature was 38.9 °C, heart rate was 97 beats per minute, respiratory rate was 24 breaths per minute, and oxygen saturation was 86% on ambient air and 94% when receiving oxygen through a nonrebreather mask at a rate of 6 liters per minute.

**Fig. 1.**
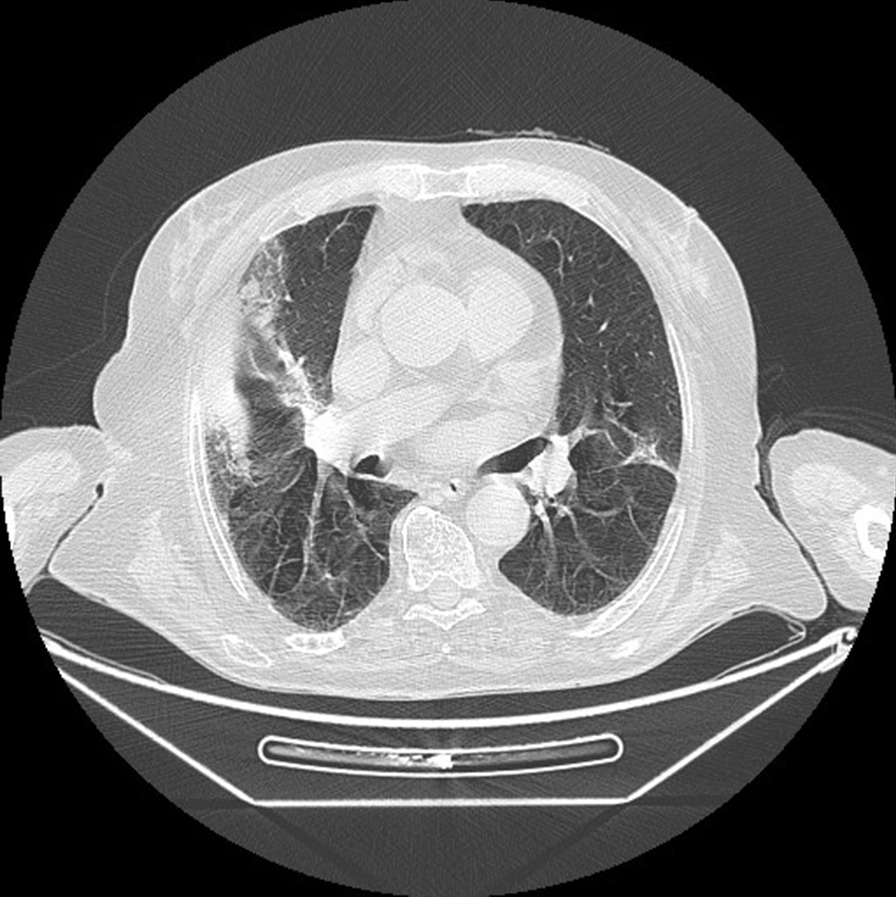
Chest computed tomography image showing ground-glass opacity, crazy paving, and consolidation

Chest radiography showed peripheral parenchymal opacities in both lungs. Chest computed tomography (CT) scan demonstrated bilateral peripheral ground-glass opacities, as well as some areas of consolidation and thickened interlobular and intralobular lines known as “crazy paving,” as shown in Fig. 1.

The patient tested positive for SARS-CoV-2 RNA and was admitted to a dedicated coronavirus intensive care unit.

Laboratory evaluation revealed high inflammatory markers: C-reactive protein 229 mg/l and serum ferritin level 1650.98 ng/ml. Hemoglobin level was 86 g/l, mean corpuscular volume 96 fl, mean corpuscular hemoglobin concentration 34.2%, and reticulocyte level 30,300/μl. White blood cell count was 5400/ml, and platelet count was 180,000/μl. D-dimer level was slightly elevated at 0.6 μg/ml, and prothrombin time was not prolonged.

Regarding treatment, during his hospitalization, the patient received oxygen, azithromycin, hydroxychloroquine, vitamin C, zinc, low-molecular-weight heparin, and corticosteroids.

Ten days after his hospitalization, there was a clinical amelioration of his symptoms including respiratory distress and fever, as well as a decrease in inflammatory markers. The patient was therefore discharged.

Two months later, he presented to the hospital for increasing dyspnea with limited physical activity, asthenia, palpitations, and tinnitus. We suspected a second infection of SARS-CoV-2; however, the result of reverse-transcription polymerase chain reaction (RT-PCR) was negative. The patient did not report any history of bleeding, as well as no fever or bone pain.

On examination, heart rate was 110 beats per minute, temperature was 36.2 °C, respiratory rate was 17 breaths per minute, and oxygen saturation was 95% on ambient air. The skin and the mucous membranes were pale with no associated jaundice. There was no hemorrhagic syndrome, no palpable peripheral lymphadenopathy, and no splenomegaly. Cardiac, pulmonary, and abdominal examinations were normal.

Chest radiography showed no opacity. Laboratory evaluation revealed normocytic normochromic anemia with reticulocytopenia. Hemoglobin level was 50 g/l, mean corpuscular volume 84.8 fl, mean corpuscular hemoglobin concentration 34.2%, and reticulocyte level 9300/μl. White blood cell count was 3400/ml, and platelet count was 202,000/μl. The peripheral blood smear showed no anomaly.

The serum ferritin level was high at 836.04 ng/ml. Regarding other inflammatory markers, the C-reactive protein level was not elevated at 10.65 mg/l.

Blood levels of total, direct bilirubin, and lactate dehydrogenase were all normal, as were the results of renal function tests. Blood levels of vitamin B12 and folate were normal, and erythropoietin level was also in the normal range.

**Fig. 2.**
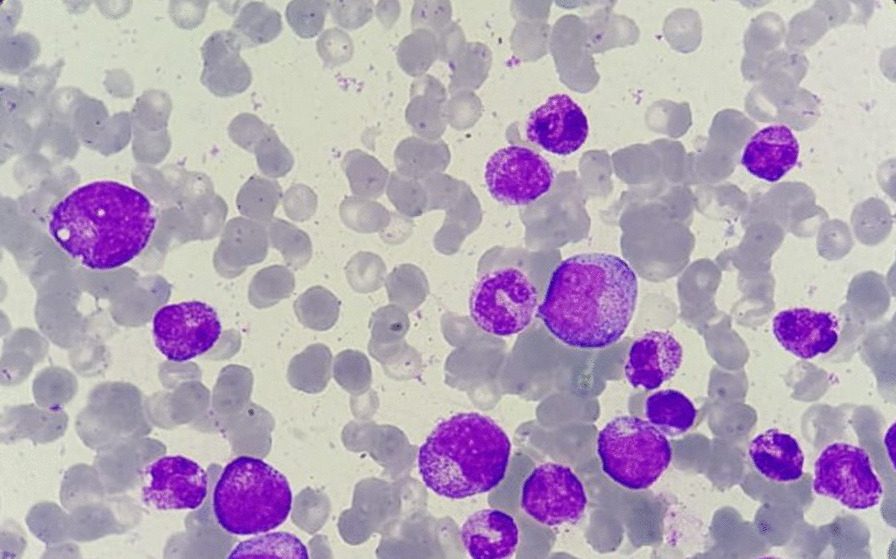
Bone marrow aspirate smear showing paucity of erythroid precursors (Giemsa, magnification ×1000, oil)

Bone marrow aspirate showed mild hypocellularity. Megakaryocytes were present and morphologically normal. The erythrocytic lineage was aplastic, accounting for less than 1%, and there were no giant pro-erythroblasts with vacuolated cytoplasm. The granulocytic lineage showed hyperplasia accounting for 82%, and all stages of maturation were present. Besides, no signs of cytological dysplasia or extramedullary cell infiltration were noted (Fig. 2)

A bone marrow biopsy was performed. Trephine biopsy sections showed normocellular bone marrow with absence of erythroid precursors, increased granulopoiesis, and normal megakaryopoiesis. There was no evidence of lymphoid or blast infiltration, no secondary deposit, and no granuloma.

The diagnosis of acquired pure red cell aplasia was retained. An etiological investigation was initiated.

The patient was not taking any drugs or chemicals such as isoniazid or azathioprine known to cause pure red cell aplasia. PCR testing for parvovirus infection was negative. Serologic tests for hepatitis A, B, and C viruses and human immunodeficiency virus were negative. Testing for antinuclear antibodies and anti-double-stranded DNA antibodies was negative. He had no history of solid cancer and no symptoms potentially related to malignant disease, and tumor markers were normal. As for radiologic findings, computed tomography of the chest, abdomen, and pelvis was normal, ruling out thymoma and deep-seated enlarged lymph nodes, which might be linked to lymphoproliferative disorders.

In conclusion, no disease associated with secondary pure red cell aplasia was detected. Oral prednisone 1 mg/kg was initiated as standard treatment. The evolution of the disease was marked by the amelioration of symptoms and a gradual increase in hemoglobin level.

## Discussion/conclusion

We report a case of acquired pure red cell aplasia following COVID-19.

Anemia is not a common finding in SARS-CoV-2 infection. This low incidence may be related to the long lifespan of erythrocytes and the compensatory production of erythrocytes caused by COVID-19-related hypoxia.

In mild COVID-19, hemoglobin might be decreased, but the mean value is usually around 13.1 g/dl as reported by Kossi *et al*. [4]. Low hemoglobin values are significantly correlated with severity and progression of COVID-19, as stated by Taneri *et al*. [5] and Cen *et al*. [6], who showed that hemoglobin levels below 11.0 g/l are related to the progression of COVID-19.

Currently, the cause of anemia in SARS-CoV-2 infection is not fully elucidated. The decrease of hemoglobin may be related to autoimmune disorders as reported by Lazarian, who described seven cases of patients with COVID-19 who developed autoimmune hemolytic anemia (AIHA) with average hemoglobin of about 70 g/l [7]. Alternatively, it may be caused by abnormal iron metabolism mediated by interleukin-6. Anemia may also be secondary to inflammation, which is due to the overproduction of pro-inflammatory cytokines.

Pure red cell aplasia is a rare disorder defined by normocytic normochromic anemia with severe reticulocytopenia and absence of erythroid precursors from the bone marrow (< 1% erythroblasts on marrow differential count). Diamond–Blackfan anemia is a frequent form of congenital PRCA that is associated with physical morphologic abnormalities. Acquired PRCA might be primary or secondary. Primary acquired PRCA is an autoimmune disorder involving an immune mechanism that interrupts normal erythroid differentiation, whereas secondary acquired PRCA is associated with underlying diseases such as autoimmune disorders, collagen vascular diseases, infections (especially parvovirus B19), lymphoproliferative disorders or other hematologic malignancies, and nonhematologic neoplasms (mainly thymoma, drugs, and toxic agents), as well as pregnancy.

The onset of acquired pure red cell aplasia weeks after recovery from acute COVID-19 suggests a delayed complication of COVID-19 related to a prolonged immune response that interrupts normal erythropoiesis. Alternatively, it might be due to the destruction of kidney tissue rich in angiotensin-converting enzyme 2 (ACE2) receptors by the virus. In fact, coronavirus is known to use ACE receptors to penetrate target cells [1], and SARS-CoV-2 has 10–20-fold greater ability to bind to the receptors compared with SARS-CoV [8]. The destruction of kidney tissue compromises erythropoietin production, and therefore reduces red cell production. However, in our case, the level of erythropoietin was not decreased, ruling out this hypothesis.

Finally, the occurrence of PRCA after COVID-19 infection might be due to coincidence.

## Data Availability

The data used to support the findings are included in the article.

## Abbreviations

SARS-CoV-2: Severe acute respiratory coronavirus 2
COVID-19: Coronavirus disease 2019
PRCA: Pure red cell aplasia
PCR: Polymerase chain reaction
